# Supplementary material for: The causal role of circulating inflammatory markers in osteoporosis: a bidirectional Mendelian randomized study
Source: Front Immunol. 2024 Jul 18;15:1412298. doi: 10.3389/fimmu.2024.1412298 (PMC11291241; doi:10.3389/fimmu.2024.1412298)
Supplement: Supplementary file 1 [file DataSheet_1.zip › supplementary material/OSTEOPOROSIS-CIM/GCST90274839.tsv.gz_leave_one_out.pdf]

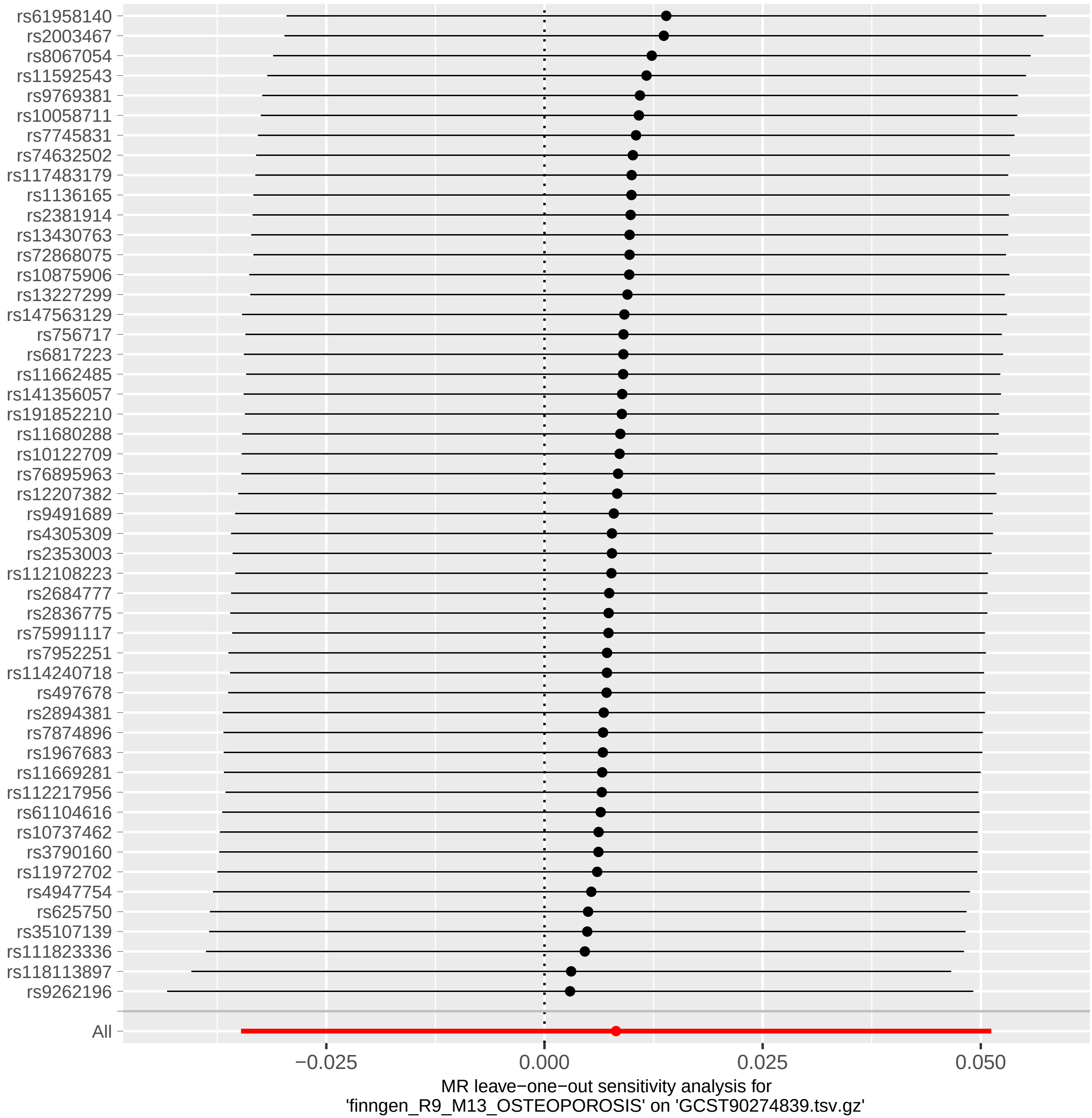
rs61958140
rs2003467
rs8067054
rs11592543
rs9769381
rs10058711
rs7745831
rs74632502
rs117483179
rs1136165
rs2381914
rs13430763
rs72868075
rs10875906
rs13227299
rs147563129
rs756717
rs6817223
rs11662485
rs141356057
rs191852210
rs11680288
rs10122709
rs76895963
rs12207382
rs9491689
rs4305309
rs2353003
rs112108223
rs2684777
rs2836775
rs75991117
rs7952251
rs114240718
rs497678
rs2894381
rs7874896
rs1967683
rs11669281
rs112217956
rs61104616
rs10737462
rs3790160
rs11972702
rs4947754
rs625750
rs35107139
rs111823336
rs118113897
rs9262196
All
−0.025
0.000
0.025
0.050
MR leave−one−out sensitivity analysis for
'finngen_R9_M13_OSTEOPOROSIS' on 'GCST90274839.tsv.gz'
